# Terrain Feature Estimation Method for a Lower Limb Exoskeleton Using Kinematic Analysis and Center of Pressure

**DOI:** 10.3390/s19204418

**Published:** 2019-10-12

**Authors:** Myounghoon Shim, Jong In Han, Ho Seon Choi, Seong Min Ha, Jung-Hoon Kim, Yoon Su Baek

**Affiliations:** 1 Motion Control Laboratory, Department of Mechanical Engineering, Yonsei University, Seoul 03722, Korea; smh2119@yonsei.ac.kr (M.S.); gmiilp1318@yonsei.ac.kr (J.I.H.); eggr@yonsei.ac.kr (H.S.C.); choosesay@yonsei.ac.kr (S.M.H.); 2 Construction Robot and Automation Laboratory, Department of Civil & Environmental Engineering, Yonsei University, Seoul 03722, Korea; junghoon@yonsei.ac.kr

**Keywords:** walking terrain, terrain estimation, terrain classification, kinematic analysis, center of pressure, lower limb exoskeleton

## Abstract

While controlling a lower limb exoskeleton providing walking assistance to wearers, the walking terrain is an important factor that should be considered for meeting performance and safety requirements. Therefore, we developed a method to estimate the slope and elevation using the contact points between the limb exoskeleton and ground. We used the center of pressure as a contact point on the ground and calculated the location of the contact points on the walking terrain based on kinematic analysis of the exoskeleton. Then, a set of contact points collected from each step during walking was modeled as the plane that represents the surface of the walking terrain through the least-square method. Finally, by comparing the normal vectors of the modeled planes for each step, features of the walking terrain were estimated. We analyzed the estimation accuracy of the proposed method through experiments on level ground, stairs, and a ramp. Classification using the estimated features showed recognition accuracy higher than 95% for all experimental motions. The proposed method approximately analyzed the movement of the exoskeleton on various terrains even though no prior information on the walking terrain was provided. The method can enable exoskeleton systems to actively assist walking in various environments.

## 1. Introduction

Wearable robots or exoskeletons, which are attached to a wearer’s body, are systems that extend, complement, substitute, or enhance the functioning and capability of the wearer by using actuators that can provide mechanical power [1]. The exoskeleton, which combines human intelligence capable of coping with various situations or circumstances and the ability of the robot to handle time-consuming simple tasks or high loads, has been developed for use mainly in military, industrial, and medical applications [2,3,4]. Among them, exoskeletons in the medical and rehabilitation fields are expected to perform as devices for enhancing the physical functions of patients weakened because of damage or aging of the nervous and musculoskeletal system [5,6]. In particular, as the lower limb exoskeleton for gait rehabilitation can restore walking ability, which is an important activity in human life, it can be a solution that will improve the quality of life of patients by enabling patients or elderly people to continue their personal and social activities.

The main function of the lower limb exoskeleton for gait rehabilitation or assistance is to recover or improve the wearer’s ability to walk, by replacing or supplementing the wearer’s leg functions. The exoskeleton is generally classified into treadmill-based and orthosis-based systems [7]. The major difference between the two systems is that the former repeats only certain actions in a limited space, while the latter can move freely without space constraints depending on the wearer’s intentions. Owing to the mobility offered by the latter system, it has attracted considerable attention for applications in activities of daily living (ADLs), including level walking. However, the development of control algorithms for effective assistance and wearer safety in a variety of unspecified conditions and terrains remains a challenging task.

The generalized control framework proposed by Tucker et al. [8] demonstrates the considerations in controlling lower limb prosthesis and orthosis (P/O) devices. They developed the framework comprising the following elements: user, P/O device, controller, and the environment. They also described the role and meaning of each element in a comprehensive and concise manner. In particular, as the environment is a significant factor influencing the stability and balance control of P/O devices, it is significantly important to consider the environment in which lower limb prostheses or exoskeletons operate outside of a laboratory in actual applications. Du et al. [9] demonstrated that prior knowledge about the operating environment is effective for improving the accuracy in classifying the locomotion mode of a powered knee. To address these requirements, Cybathlon 2020 [10], which is a unique world championship for people with physical disabilities, is proposing various terrains, such as rough terrains, stairs, tilted paths, and ramps, to challenge exoskeleton systems. Therefore, to provide efficient and stable assistance to wearers of exoskeletons in various environments in daily life, it is necessary to consider various types of operating environments.

Terrain, which is an element of the environment and corresponds to the geometry of the ground, is a key factor influencing the safety of the wearer requiring walking assistance from the lower limb exoskeleton. To control the exoskeleton properly according to the various terrains, the information of a standing terrain should be identified. There are two methods for identifying terrains: explicit methods, which measure the geometry of terrains directly using additional sensors, and implicit methods, which estimate the terrain geometry using sensors that are embedded in the exoskeleton [8]. The former uses a laser distance meter [11], camera [12], or an infrared range sensor [13,14] to measure the terrain features in front of the wearer. The latter uses an inertial measurement unit (IMU) [15,16,17] or an electromyography (EMG) sensor [18,19] to classify the types of terrains on which the wearers will walk in their current or next steps. The explicit method may be more suitable for controlling the lower limb exoskeleton than the implicit method in that it can estimate the terrain that the wearer would walk on in the next step; however, utilizing additional sensors can increase the burden on the controller and increase the cost of the system. On the other hand, the implicit method can estimate information about the current terrain only; however, it can be compensated by using the characteristics of walking movements, which are repetitive and cyclic [20], and algorithms that compute the intention of a wearer’s walking. Moreover, because this method does not use additional sensors but uses built-in sensors for control in the majority of exoskeleton systems, it can be used freely in most exoskeletons.

Therefore, in this study, we developed a terrain feature estimation method that can be applied for controlling the lower limb exoskeleton for effective walking assistance and safety of the wearer on various walking terrains. The proposed method utilizes the center of pressure (CoP), which is measured by the foot pressure sensor, as the contact point between the exoskeleton and the ground and calculates the position of the contact point in space through kinematic analysis. Because all these contact points created during walking are points on the ground, the geometry of the ground can be determined through the trajectories of these points. The contact point set calculated for each step is modeled as a plane that reflects the geometry of the ground by using the least-square method. Finally, the proposed method estimates the slope and elevation of the terrain for each step by using the normal vector of the modeled plane. Unlike previous studies that focused on the type of terrain for selecting the locomotion mode, our study focused on understanding the detailed features of terrains, such as the slope, and elevation, to provide further information for control algorithms. Furthermore, as our method does not utilize any additional sensors other than posture, angle, and foot sensors, which are used in general exoskeletons, to control the system and to identify the wearer’s intention, it can be easily configured in a variety of exoskeleton systems and will not affect the cost of the system.

In the lower limb exoskeleton used in this study, the hip and knee joints were assisted by an electrical actuator that was configured as a module. Modular actuators can be selectively attached to or detached from joints that require assistance depending on the wearer’s condition or purpose. This allows the exoskeleton to be operated in different modes depending on the situation and can thus be applied to various subjects. We describe the exoskeleton used in developing the terrain feature estimation method in the next section.

The remainder of the paper is organized as follows: Section 2 is comprised of the descriptions for the materials and methods that are used for the development in this study. The first portion of Section 2 describes the mechanical structure, sensor system, and modularization of the actuation part of the developed lower limb exoskeleton. The second portion of Section 2 describes the calculation of the spatial position of the CoP through kinematic analysis of the exoskeleton and terrain feature estimation using this spatial position. Section 3 describes the experimental results for level ground, stairs, and a ramp using the developed method. Section 4 presents the discussion of the results and concludes the paper.

## 2. Materials and Methods

### 2.1. Overall Structure of the Exoskeleton

**Figure 1:**
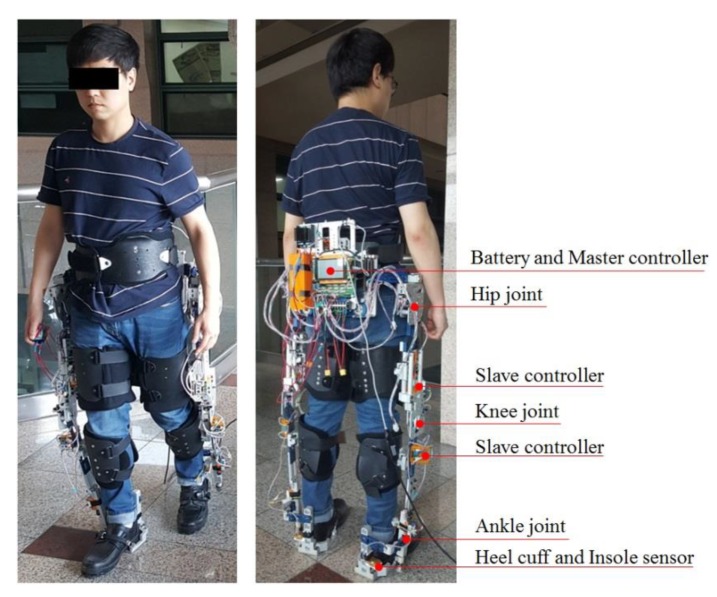
Subject wearing the lower limb exoskeleton and main configuration of the system.

The exoskeleton system illustrated in Figure 1 was designed for normal people and for patients or elderly individuals with partially weakened muscles due to nervous system diseases or aging. The system is comprises orientation and angular sensors for calculating its spatial posture, force sensors inserted in the fastening parts between the wearer and robot for detecting wearer’s intention. And electric motors are used to support motion of the hip and knee joints of a wearer during walking, sit-to-standing and squatting in their daily lives or rehabilitation trainings by decreasing the load on the joints. In this study, we focused on estimating the normal vector of the ground surface on which the wearer stands through kinematic analysis of the exoskeleton; therefore, actuator control was not considered. However, we briefly describe all the components of the exoskeleton including the actuators in the following sections.

#### 2.1.1. Mechanical Joints and Components

Each leg of the exoskeleton was designed to accommodate the motion of human joints in the sagittal, transversal, and frontal planes, which are responsible for stretching the legs forward to advance, changing walking direction by rotating legs, and maintaining balance by shifting the weight center, respectively. The leg has three joints, namely the hip, knee, and ankle joints; they were positioned to reduce discomfort caused by misalignments between the exoskeleton and wearer by aligning their axes to pass through the anatomical joint axis of the wearer [21].

**Figure 2:**
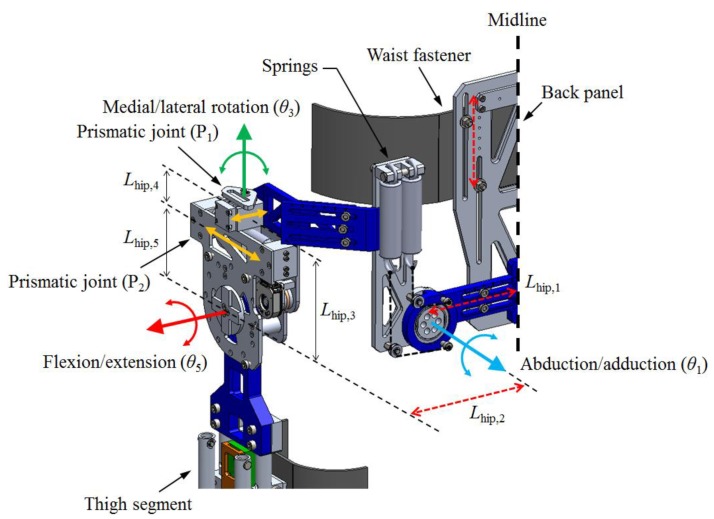
Hip joint structure for hip flexion/extension, abduction/adduction, and medial/lateral rotation.

**Figure 3:**
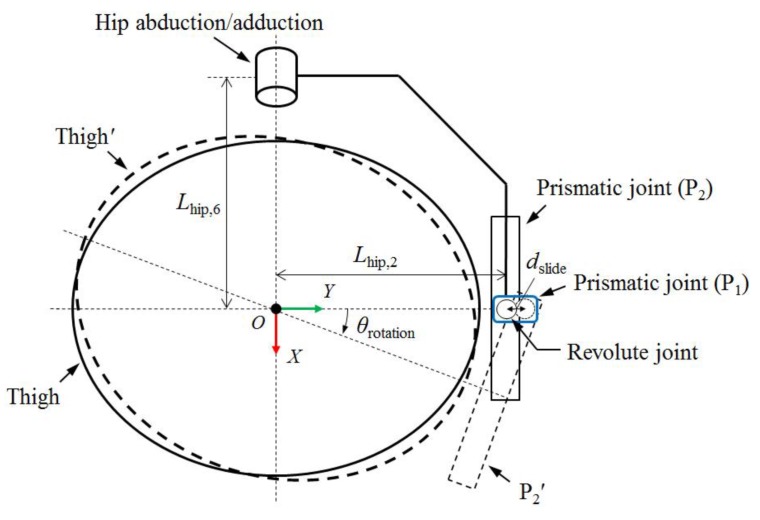
Schematic of the remote-center rotation of hip medial/lateral joint in the transversal view.

The hip joint of the exoskeleton has five degrees of freedom (DOFs). The joint is composed of two revolute joints for hip flexion/extension and adduction/abduction and one revolute with two prismatic joints for hip medial/lateral rotation (Figure 2). The axes of the first two joints can be easily aligned to pass through the center of the hip joint, which is usually modeled as a ball and socket joint [22], by adjusting the location of the axes manually. However, as the axis of hip medial/lateral rotation is inside the wearer’s hip joint and thigh segment, the remote-center rotation mechanism comprising linkages [23,24] or curved sliders [25,26] is required to align the axis of the exoskeleton to the axis of the wearer’s hip motion. It is because the motion of a rigid body rotating about a remote-center consists of rotation and translation; these mechanisms cause the axis of the revolute joint to slide on the transversal plane during hip medial/lateral rotation. For this reason, we added two prismatic joints (P_1_, P_2_) to the hip medial/lateral rotation joint to align the axes of the exoskeleton and wearer (Figure 3). P_1_ (blue rectangle) is fixed to the link attached to the hip abduction/adduction joint. The remaining part of each leg is connected to P_2_. The revolute joint for the hip medial/lateral rotation is located between P_1_ and P_2_. The prime symbol (′) denotes the position of the thigh segment and remaining part of the leg after medial/lateral rotation (*θ*_rotation_). By allowing movement (*d*_slide_) of the revolute joint with P_1_, the rest of the leg can rotate with a constant distance (*L*_hip,2_) from the center. Inside the prismatic joints, springs were added to maintain a neutral position. Moreover, springs were installed in the hip adduction/abduction joint to compensate for the torque caused by the weight of the exoskeleton leg.

**Figure 4:**
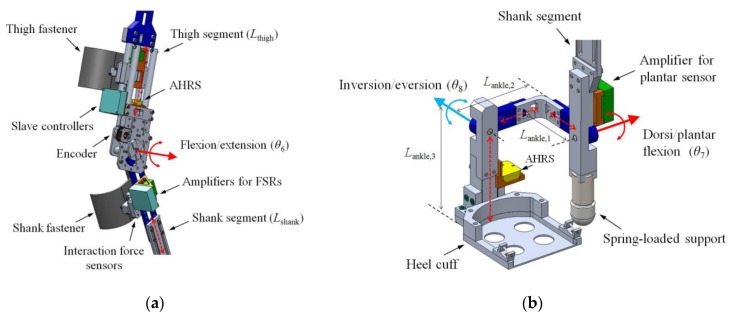
Configuration of (**a**) the joint for knee flexion/extension and (**b**) the joint for ankle dorsi/plantar flexion and inversion/eversion.

The knee joint of the exoskeleton has a one DOF revolute joint for knee flexion/extension (Figure 4a). Technically, a human knee joint cannot be modeled as a simple hinge joint because it shows polycentric motion, i.e., its instant center of rotation is not fixed [27]. However, as the variation of the axis in walking is relatively small (approximately 10–15 mm [28,29]), it could be compensated by the motion of the other joints. Thus, we designed the knee joint as a revolute joint.

The ankle joint of the exoskeleton is composed of two revolute joints for ankle dorsi/plantar flexion and inversion/eversion (Figure 4b), which occur concurrently during walking [30]. We did not consider the rotation of the ankle joint on the transversal plane because it mainly resulted from the hip medial/lateral rotation. A spring-loaded support that transfers the weight of the exoskeleton to the ground was added under the ankle dorsi/plantar flexion joint to reduce the burden of the wearer.

**Figure 5:**
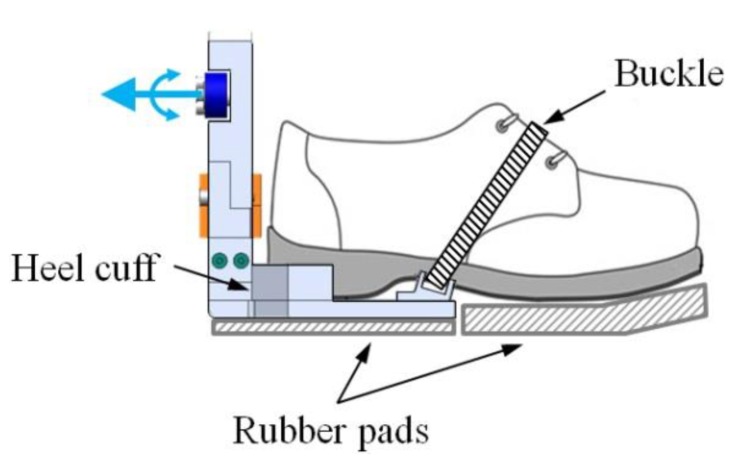
Foot configuration of the exoskeleton. The forefoot is free to be bent for ensuring smooth ground contact.

The foot segment of the exoskeleton secures the foot of the wearer wearing a shoe by using a buckle and covers only the heel side of the wearer’s foot (Figure 5). This configuration permits the forefoot to be bent during the terminal stance phase. It is effective for walking naturally [31,32] because the flexibility of the foot required for smooth contact is maintained. Rubber pads are attached under the sole of the foot segment and the wearer’s shoe for shock absorption and to prevent slip, and to compensate for the height difference between the wearer’s shoes.

The available ranges of motion (ROMs) of each joint were set to be between the total ROMs of anatomical human joints and the ROMs of normal walking motions (Table 1). For wearer safety, mechanical stoppers were installed to restrict the joints so that they did not exceed their allowable ROMs.

The frames were made of an aluminum alloy (7075 used for high-stress parts, 6061 used for the other parts) for obtaining a light-weight structure. The length of each link is manually adjustable for adapting to different body sizes of wearers by using a slider and a bolt with a spring washer. The adjustable ranges of each link length were set based on the dataset of Korean males aged 20 to 60 years [33]. The physical interface on the back, thigh, and shank segment was composed of a rigid cuff with a soft pad and Velcro straps. It covers a large area of the wearer’s body to prevent pressure concentration.

#### 2.1.2. Sensors and Electronics

**Figure 6:**
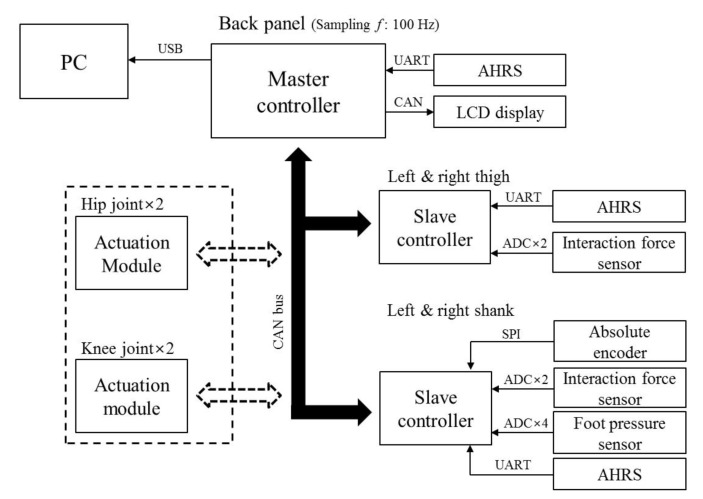
Schematic of the control system.

The schematic of the entire control system is illustrated in Figure 6. We designed a distributed controller architecture to reduce the calculation load caused by a large amount of sensor data on the master controller (180 MHz, 32F429I-DISC1, STMicroelectronics). The slave controllers (72 MHz, NUCLEO-F303K8, STMicroelectronics) were installed on the thigh and shank segment of each leg, respectively; they collected the data measured by the sensors on each segment. The collected data of each slave controller were transferred to the master controller through the controller area network (CAN) protocol. An attitude and heading reference system (AHRS; 3DM-GX4-25, LORD-MicroStrain^®^) was installed on the back panel and was directly connected to the master controller. The master controller on the back panel merged the data from the slave controllers and the acquisition time into a unified dataset. An LCD mounted board (180 MHz, 32F469IDISCOVERY, STMicroelectronics) was stacked above the master controller for monitoring the acquired dataset, and a PC was used for recoding the dataset. The acquisition rate of the dataset was set to 100 Hz. Two Li–Po batteries (11.1 V, 2600 mAh) with a 5 V regulator were utilized to supply power to the controllers and sensors. An emergency switch was utilized for the safety of the wearer during the test.

To estimate the spatial position and orientation of the exoskeleton, a total of five AHRSs and two absolute encoders were utilized to measure the angle of each joint. In many studies, for controlling an object in space, it is common to use an AHRS or an IMU. However, these require a magnetometer and carefully designed filter to correct accumulated errors such as drifts. Moreover, they have a relatively lower resolution than encoders; thus, measuring a joint angle using an AHRS or IMU is considered inappropriate in robotics. Nevertheless, several studies [34,35] estimated the joint angle of a manipulator with AHRSs and IMUs to utilize their advantages of contactless sensing and simple installation. Additionally, as an AHRS returns the attitude as an Euler angle consisting of three independent variables (roll, pitch, and yaw), it can estimate up to three joint angles without installing encoders on all the joint axes to be measured [36,37]. Thus, we applied two additional AHRSs (MW-AHRSv1, NTREX Corp.) on each leg to estimate the angle of the hip and ankle joint, which have multiple DOF structures. The angle of the knee joint, which has one DOF, was measured by an absolute encoder (12-bit resolution, AMT203, CUI). Consequently, the number of sensors required for estimating the state of each leg of the exoskeleton, which six DOFs, was reduced to three from six, which was required when using encoders.

Unfortunately, in this study, it is impossible to utilize a magnetometer for compensating the yaw drift because of the magnetic disturbance caused by electrical actuators and the indoor environment. Thus, we used only the gyro measurements for calculating yaw angles, and tests using the system were conducted for short durations to minimize the drift error. The level of the drift error was checked up to ±0.1 °/min in a stationary condition.

**Figure 7:**
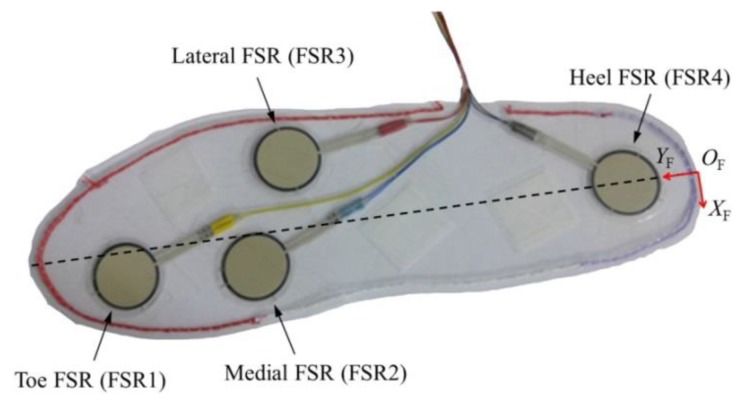
Insole sensor for measuring plantar pressure distribution.

Thin and flexible force sensing resistors (FSRs) were used to measure the foot plantar pressure distribution and the interaction forces exerted through the physical interface on the wearer’s thigh and shank. Our previous study [38] has shown that measuring these forces is useful for the exoskeleton to recognize the wearer’s intention and to operate in accordance with it. To insert the FSRs into the shoes of the wearer, an insole-type sensor was fabricated by bonding four FSRs (A401, Tekscan Inc.) to a polypropylene sheet (Figure 7). The FSRs were placed on protruding areas of the human foot where pressure concentrates during walking [39,40]. The interaction forces were also measured using FSRs (A301, Tekscan Inc.) that were smaller than the FSRs on the insole sensor.

**Figure 8:**
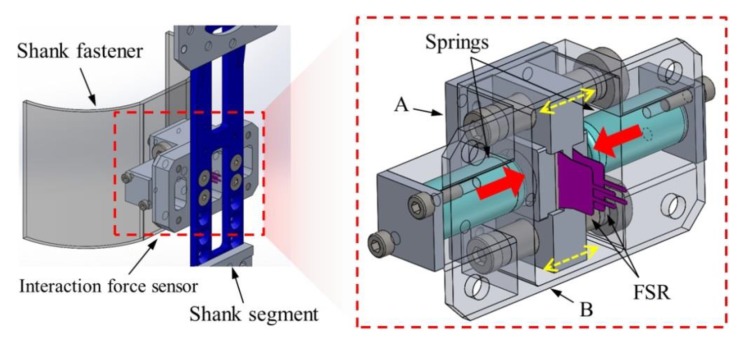
Custom-made sensor for measuring the interaction forces on the thigh and shank segments.

**Figure 9:**
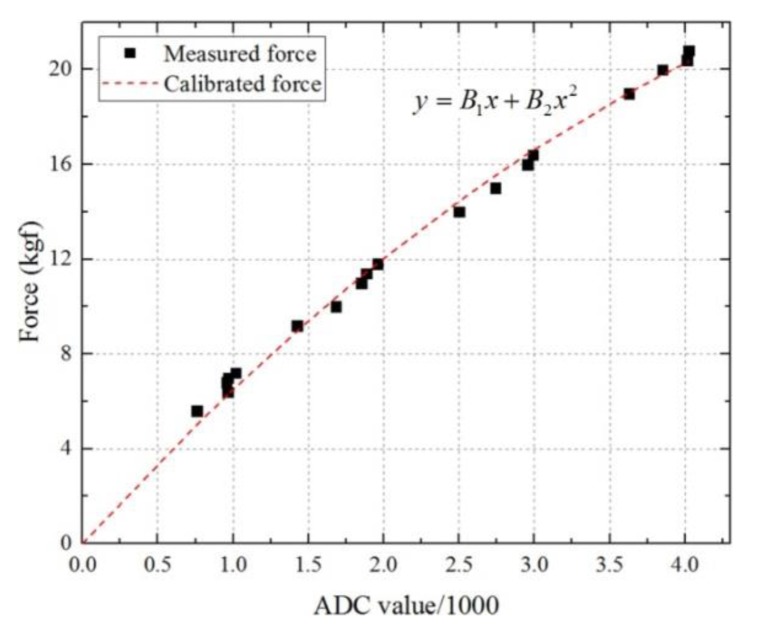
Calibration curve of the FSR in the range of 0 to 20 kgf. The ADC values divided by 1000 were fitted with a second order polynomial. The coefficients of this curve, B_1_ and B_2_, are 6.97 and −0.47, respectively.

Custom-made sensors (Figure 8) for measuring the interaction forces between the wearer and exoskeleton were installed on the base of each fastener of the thigh and shank segments. As an FSR can only measure compressive forces, a pair of FSRs was inserted with springs to measure bidirectional forces. Parts A and B shown in Figure 8 were fixed to the fastener (wearer side) and the thigh or shank frame (exoskeleton side), respectively. When the wearer moves his/her limb, part A is translated along the linear guides (yellow arrows) and compresses the spring. Then, the measured force (red arrows) on one of the FSR will be increased while that on the other side will be decreased. All of the FSR output signals were filtered using a second order Butterworth filter with a 10 Hz cutoff frequency. The output of each FSR was calibrated with a second order polynomial curve for the range of 0–20 kgf (Figure 9).

#### 2.1.3. Actuation Modules

**Figure 10:**
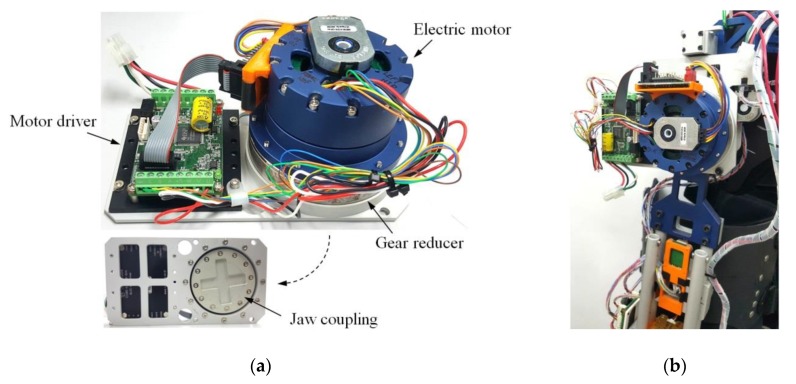
(**a**) Composition of the actuation module and (**b**) the assembly state with the hip joint of the exoskeleton.

**Figure 11:**
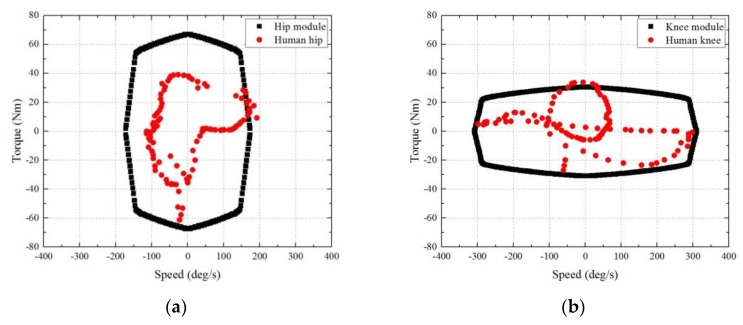
Comparison for (**a**) hip and (**b**) knee torque–speed characteristics of the actuation module and human joint during walking. The angular speed of each joint was calculated based on the stride time of 1.1 s.

The flexion/extension of the hip and knee joints was assisted by the actuation module shown in Figure 10. Unlike built-in actuators of a general exoskeleton, the actuation modules designed in this study can be detached from the exoskeleton frame and selectively provide assistive torque to the joints. Modularization can be one of the means to extend the capability of an exoskeleton limited to a specific user or task. Through modularization, a system can be reconfigured depending on the user requirements [41], applying a specific stiffness or damping force on the joint [42] and converting from a passive orthosis to a motorized orthosis [43]. Consequently, it will offer different testing options for developers and provide various functions for users and reduce the cost for customers. The actuation module is composed of an electric motor, a gear reducer (100:1 for hip module and 80:1 for knee module, SHD-20-2SH, Harmonic Drive LLC), and a motor driver (24V-16A, CUBE-2416-SIH, Robocube Tech co.) with the CAN protocol. Two types of electric motors (TBMS-6025-B for hip module and TBMS-6013-B for knee module, KOLLMORGEN) were selected to handle the torque–speed characteristics of human hip and knee joint during walking [44], as shown in Figure 11. Jaw couplings were applied to the output shaft of the module and joint of the exoskeleton to transfer the output power of the actuator to the wearer’s joint.

**Figure 12:**
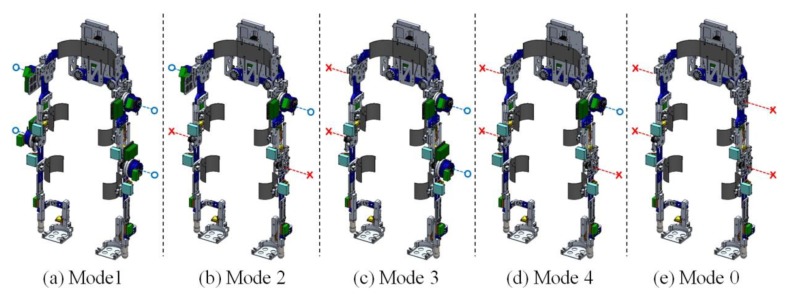
Configuration of the five modes of the exoskeleton according to the assembly of the actuation modules. The blue-circle and red-cross indicates whether the actuation module is assembled to the joint or not, respectively. Each mode of the exoskeleton can be used for the purpose of assisting (**a**) hip and knee joints of both legs; (**b**) hip or knee joints of both legs; (**c**) hip and knee joints of one leg; and (**d**) one of the joint of lower-limb. In the case of (**e**) mode 0, although it cannot assist the joints of a wearer by using actuation modules, it can be used to measure the motion of a wearer by using embedded sensors.

The exoskeleton developed in this study can operate in five different modes through assembling the actuation modules depending on the assistance requirements of wearers, as shown in Figure 12. If a wearer cannot move his/her lower limb entirely, mode 1, with four actuation modules on the hip and knee joints, is appropriate for assistance. Likewise, the other modes can be used according to the assistance requirements of wearers. In mode 2, actuator modules are attached to both the hip or knee joints; in mode 3, the actuation modules are attached to the hip and knee joints of one of the legs; in mode 4, the actuator modules are attached to one of the lower limb joints. Although the exoskeleton in mode 0 where no actuation module is incorporated cannot support the movement of the wearer, the joint angle and plantar pressure data of the wearer can be obtained from sensor measurements thus it is available to be utilized as a motion analysis device. In this study, we utilized the exoskeleton with mode 0 in developing the terrain feature estimation method because this mode can follow the wearer’s movements without using additional control schemes such as the transparent mode [45] to eliminate the actuator inertia. The total weight of the exoskeleton with and without the actuation modules is 9.5 kg and 15.5 kg, respectively.

### 2.2. Strategy for Terrain Feature Estimation

This study calculated the position of the contact points on the ground at each step while the wearer walked; the slope and elevation of the terrain were estimated by modeling the contact points as a spatial plane. The proposed method calculated the position and orientation of each segment and foot through kinematic data derived from the embedded sensors. Simultaneously, we used the plantar pressure, which was measured by the insole sensor, to calculate the CoP and utilized it as a contact point with the ground. This was then introduced into the previously performed kinematic analysis, and the spatial locations of the contact points were collected while walking. The collected contact points on the ground on which the exoskeleton stepped were modeled as a plane through the least-square method. Finally, we used the normal vectors of the modeled planes during each step to estimate the terrain features, namely the slope and elevation.

#### 2.2.1. Kinematic Analysis of Lower Limb Exoskeleton

**Figure 13:**
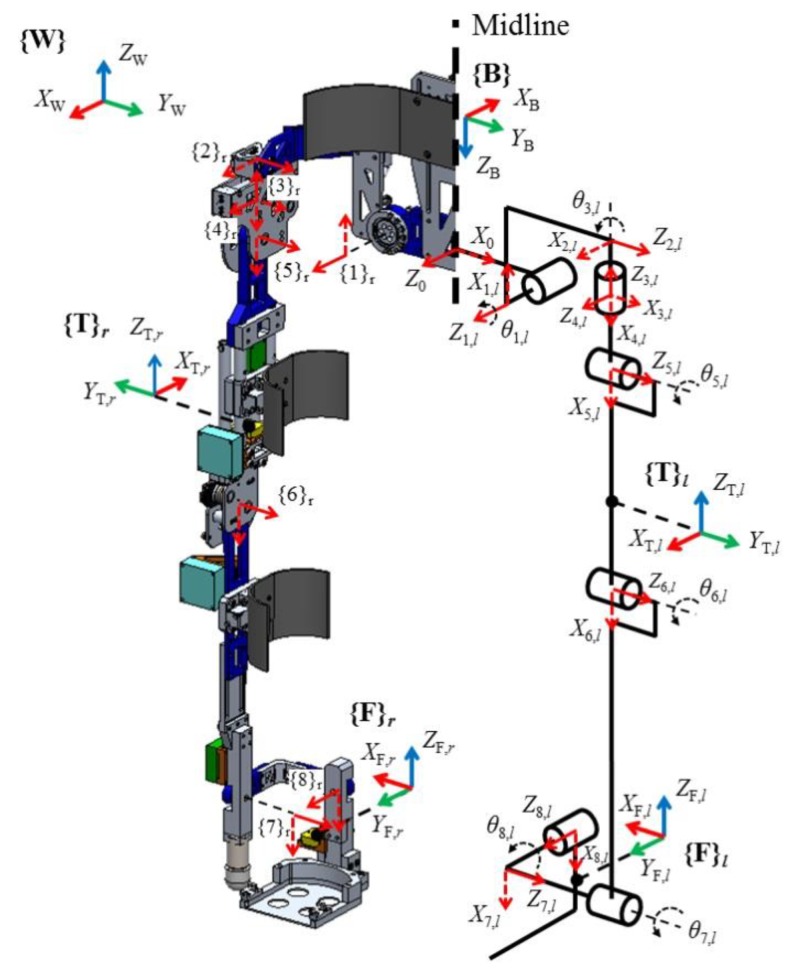
Kinematic model of the exoskeleton and its frame attachment.

Kinematic analysis of the lower limb exoskeleton was performed based on the Denavit–Hartenberg (D–H) convention [46]. Starting from the back panel, frame {0}, the frames consisting of the X (dotted red arrow) – Z (solid red arrow) axis were sequentially attached to the joints of each leg with eight DOFs, as shown in Figure 13. X_*N*_ and Z_*N*_ denote the X and Z axes of the frame attached to the *N*th segment, respectively. Furthermore, to distinguish the left and right leg frames, “r” and “l” for indicating left and right legs, respectively, are used after the frame number *N*. Although the figure shows only the kinematic model for the left leg of the exoskeleton, the frames are attached to the right leg using the same rule. In the figure frames ({B}, {T}_l,r_, and {F}_l,r_), which comprise the X–Y–Z axes, indicate the positions and postures of the AHRSs installed on the back panel, the thighs, and feet of the left and right legs. Frame {W} indicates the world coordinate system, and its Z_*W*_ axis is parallel to the vector of gravity. Table 2 presents the D–H parameters that were calculated using the defined frames. The displacement of the prismatic joints P_1_ and P_2_ was not considered because it is normally small and the joints maintain their neutral position with the help of the springs if not in an abnormal situation. The frames {2} and {4} are the virtual frames added to arrange the joints to fit the hip structure of the exoskeleton.

The homogeneous transformation matrix, ${}_{i}^{i-1}T$, which represents the orientation and position of the *i*th frame with respect to the *i*-1th frame according to the D–H convention, can be expressed as follows:

(1) $${}_{i}^{i-1}T_{l,r}={\left[\begin{array}{cc} {}_{i}^{i-1}R_{3\times 3} & {}^{i-1}d_{3\times 1}\\ 0_{1\times 3} & 1 \end{array}\right]}_{l,r}={\left[\begin{array}{cccc} c\theta_{i} & -s\theta_{i} & 0 & a_{i-1}\\ s\theta_{i}\cdot c\alpha_{i-1} & c\theta_{i}\cdot c\alpha_{i-1} & -s\alpha_{i-1} & -s\alpha_{i-1}\cdot d_{i}\\ s\theta_{i}\cdot s\alpha_{i-1} & c\theta_{i}\cdot s\alpha_{i-1} & c\alpha_{i-1} & c\alpha_{i-1}\cdot d_{i}\\ 0 & 0 & 0 & 1 \end{array}\right]}_{l,r},$$

where ${}_{i}^{i-1}R_{3\times 3}$ and ${}_{i}^{i-1}d_{3\times 1}$ respectively denote the rotation and translation parts of the transformation matrix; ‘c’ and ‘s’ denote cosine and sine functions, respectively.

The position and orientation of the foot segment and frame {8} with respect to frame {0}, which is the frame on the back panel, were calculated as follows according to the segment connection order of the exoskeleton:

(2) $${}_{8}^{0}T_{l,r}=\prod_{i=1}^{8}{}_{i}^{i-1}T_{l,r},$$

Consequently, using the rotation matrix of frame {B}, which was calculated by the AHRS installed on the back panel, the position on frame {W} of each segment was calculated as follows:

(3) $${}_{8}^{W}T_{l,r}={}_{B}^{W}T\cdot {}_{0}^{B}T\cdot {}_{8}^{0}T_{l,r},$$

where ${}_{B}^{W}T$ is calculated by the AHRS on the back panel; ${}_{0}^{B}T$ is a constant matrix determined by the installation of the AHRS on the back panel.

In this study, as explained in Section 2, the angles of the hip joint (*θ*_1_, *θ*_3_, and *θ*_5_) and ankle joint (*θ*_7_ and *θ*_8_) of both the legs, excluding the knee joint angle (*θ*_6_), were indirectly measured by the five installed AHRSs. Each AHRS returns its posture as a rotation matrix with respect to frame {W}. The following relationship was derived using frame {B} of the AHRS, which was installed on the back panel, and frame {T}_l, r_ of the AHRS, which was installed on the thigh segments:

(4) $${}_{T}^{W}R_{l,r}={}_{B}^{W}R\cdot {}_{0}^{B}R\cdot {}_{5}^{0}R_{l,r}\cdot {}_{T}^{5}R_{l,r},$$

where *R* means the rotation part of the transformation matrix *T*; ${}_{T}^{5}R_{l,r}$ is a constant matrix determined by the installation of the AHRS on the thigh segment.

As, matrix ${}_{T}^{W}R_{l,r}$ is directly measured by the AHRSs installed on the thigh segment, matrix ${}_{5}^{0}R_{l,r}$ was calculated using the known values as follows:

(5) $${}_{5}^{0}R_{l,r}={\left({}_{0}^{B}R\right)}^{-1}\cdot {\left({}_{B}^{W}R\right)}^{-1}\cdot {}_{T}^{W}R_{l,r}\cdot {\left({}_{T}^{5}R_{l,r}\right)}^{-1}={\left[\begin{array}{ccc} r_{11} & r_{12} & r_{13}\\ r_{21} & r_{22} & r_{23}\\ r_{31} & r_{32} & r_{33} \end{array}\right]}_{l,r},$$

where *r_ij_* is an element of matrix ${}_{5}^{0}R_{l,r}$.

Rotation matrix ${}_{5}^{0}R_{l,r}$ was also derived using D–H parameters in Table 2 as follows:

(6) $${}_{5}^{0}R_{l,r}=\prod_{i=1}^{5}{}_{i}^{i-1}R_{l,r}={\left[\begin{array}{ccc} s\theta_{1}c\theta_{5}-s\theta_{3}s\theta_{5}c\theta_{1} & -s\theta_{1}s\theta_{5}-s\theta_{3}c\theta_{1}c\theta_{5} & c\theta_{1}c\theta_{3}\\ -c\theta_{1}c\theta_{5}-s\theta_{1}s\theta_{3}s\theta_{5} & s\theta_{5}c\theta_{1}-s\theta_{1}s\theta_{3}c\theta_{5} & s\theta_{1}c\theta_{3}\\ -s\theta_{5}c\theta_{3} & -c\theta_{3}c\theta_{5} & -s\theta_{3} \end{array}\right]}_{l,r},$$

Therefore, the hip joint angles (*θ*_1_, *θ*_3_, and *θ*_5_) were calculated using Equations (5) and (6) as follows:

(7) $$\begin{array}{cc} \begin{array}{l} \theta_{3}=\mathrm{atan}2(-r_{33},\sqrt{r_{31}^{2}+r_{32}^{2}})\\ \theta_{5}=\mathrm{atan}2(-r_{31}/c\theta_{3},-r_{32}/c\theta_{3})\\ \theta_{1}=\mathrm{atan}2(r_{23}/c\theta_{3},r_{13}/c\theta_{3}) \end{array} & \mathrm{for}\;l,r \end{array},$$

Similarly, the ankle joint angles (*θ*_7_ and *θ*_8_) were also obtained as in Equations (4)–(6). Using frame {T}_l,r_ of the AHRS in the thigh segment and frame {F}_l,r_ of the AHRS in the foot segment, matrix ${}_{8}^{6}R_{l,r}$ was calculated as follows:

(8) $${}_{8}^{6}R_{l,r}={\left({}_{6}^{5}R_{l,r}\right)}^{-1}\cdot {\left({}_{5}^{T}R_{l,r}\right)}^{-1}\cdot {\left({}_{T}^{W}R_{l,r}\right)}^{-1}\cdot {}_{F}^{W}R_{l,r}\cdot {\left({}_{F}^{8}R_{l,r}\right)}^{-1}={\left[\begin{array}{ccc} q_{11} & q_{12} & q_{13}\\ q_{21} & q_{22} & q_{23}\\ q_{31} & q_{32} & q_{33} \end{array}\right]}_{l,r},$$

where ${}_{F}^{8}R_{l,r}$ is a constant matrix determined by the installation of the AHRS on the foot segment; matrix ${}_{F}^{W}R_{l,r}$ was calculated by the AHRS installed on the foot segment; matrix ${}_{6}^{5}R_{l,r}$ was calculated using the knee joint angle (*θ*_6_) and *q_ij_* is an element of matrix ${}_{8}^{6}R_{l,r}$.

Matrix ${}_{8}^{6}R_{l,r}$ was also derived using D–H parameters as follows:

(9) $${}_{8}^{6}R_{l,r}={\prod_{i=7}^{8}{}_{i}^{i-1}R_{l,r}}_{l,r}={\left[\begin{array}{ccc} c\theta_{7}c\theta_{8} & -c\theta_{7}s\theta_{8} & s\theta_{7}\\ s\theta_{7}c\theta_{8} & -s\theta_{7}s\theta_{8} & -c\theta_{7}\\ s\theta_{8} & c\theta_{8} & 0 \end{array}\right]}_{l,r},$$

Finally, the ankle joint angles, *θ*_7_ and *θ*_8_, of both legs were derived using Equations (8) and (9) as follows:

(10) $$\begin{array}{cc} \begin{array}{l} \theta_{7}=\mathrm{atan}2(q_{13},-q_{23})\\ \theta_{8}=\mathrm{atan}2(q_{31},q_{32}) \end{array} & \mathrm{for}\;l,r \end{array},$$

**Figure 14:**
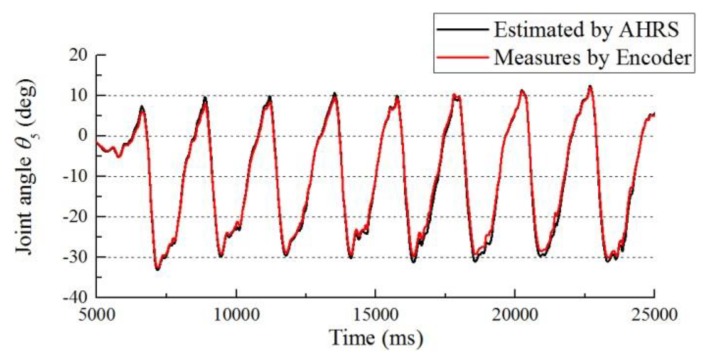
Joint angle estimation result of *θ*_5_ compared with the angle measured by an encoder.

Figure 14 compares the estimated result using Equation (7) for the hip joint angle (*θ*_5_) and the measured result using an absolute encoder.

#### 2.2.2. Calculation of CoP and Foot Phase

In this study, we used the CoP, which was calculated by using plantar pressures measured by an insole sensor (Figure 7) comprising four FSRs, and angular velocity ($\omega_{F}$) of the foot segment, measured by the AHRS, to determine the foot phase of the exoskeleton. We assumed that slip does not occur owing to the rubber pads under the sole of the foot segment by the rubber pads under the sole of the foot segment while the exoskeleton is in contact with the ground. The CoP values of both feet were calculated using the position ($r_{\mathrm{FSR},i}$) and measured force ($F_{\mathrm{FSR},i}$) of each FSR as follows:

(11) $$\mathrm{CoP}(x,y)=\frac{\sum_{i=1}^{4}r_{\mathrm{FSR},i}\cdot F_{\mathrm{FSR},i}}{\sum_{i=1}^{4}F_{\mathrm{FSR},i}};$$

the notations for the FSR and coordinate axes for the CoP are depicted in Figure 7, and the coordinate of the FSR is calculated on frame {8}_*l,r*_.

**Figure 15:**
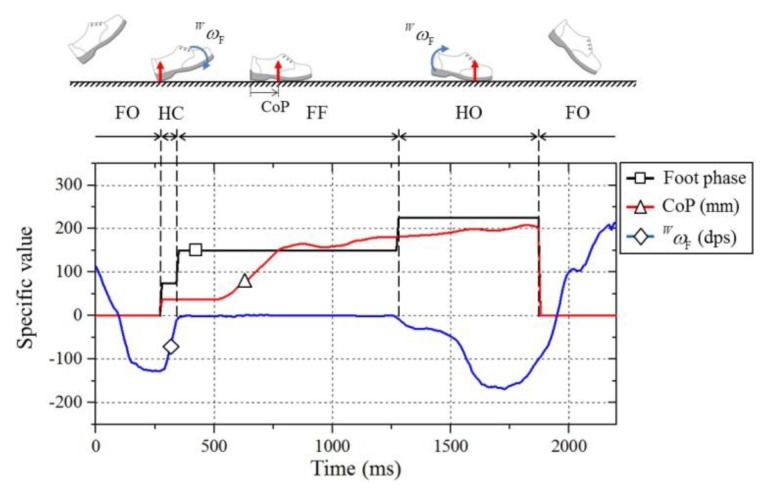
Foot contact phase determination result using the CoP and the angular velocity of the foot (*ω*_F_) with respect to frame {W}.

The foot phases were classified into four phases according to the calculated position of the CoP and angular velocity ($\omega_{F}$): heel contact (HC), foot flat (FF), heel off (HO), and foot off (FO). First, if the calculated CoP was zero, it indicated that the exoskeleton did not touch the ground. In this case, the foot phase became the FO phase. If the CoP was not zero, the foot phase was determined by considering the position of the CoP and angular velocity. If the CoP was located close to the heel side and the angular velocity was higher than a certain threshold, the foot phase became the HC phase. If the CoP was located on the front side and the angular velocity was higher than a certain threshold, the foot phase became the HO phase. In other cases, if the CoP was located in the middle of the foot or the angular velocity was low enough, the foot phase became the FF phase. Figure 15 shows the foot phase calculation results for one gait cycle while walking.

#### 2.2.3. Position Calculation in Frame {W}

The spatial movement of the exoskeleton was analyzed by calculating the relative motion of each segment with respect to the contact point on the ground being a pivot. For the analysis, it was important to choose a pivot appropriately based on foot contact conditions. In a single stance, as only the CoP for one foot, which was touching the ground, was measured, the CoP of the supporting leg was selected as the pivot. In the double stance where both legs touched the ground, the CoP of one of the feet in the FF phase, considered to be in full contact with the ground, was selected as the pivot. If both the feet were in the FF phase or any foot was not in the FF phase, the CoP on the side with more weight was selected as the pivot.

**Figure 16:**
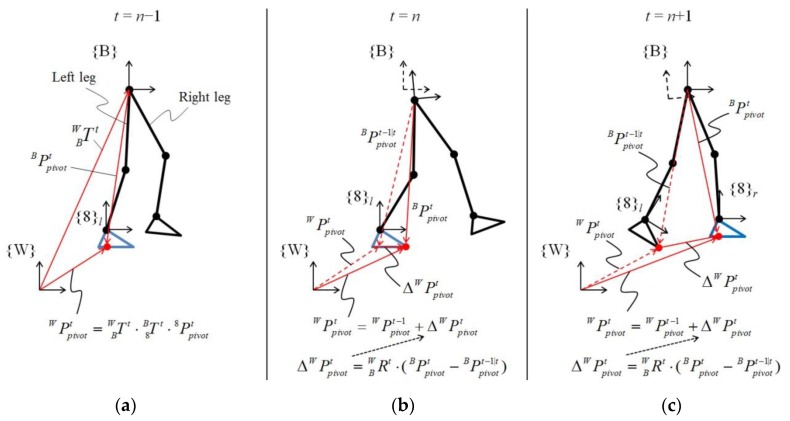
Calculation of the pivot vector on frame {W} according to the contact phase of feet. (**a**) At the first contact of a foot with the ground, the CoP of the supporting leg is set to the pivot. (**b**) As the CoP moves during foot flat phase, the pivot is continuously updated for the variation of the CoP. (**c**) When the supporting leg is changed to the other leg, the position vector between both feet is used to update the pivot.

Figure 16 shows the calculation of the position vector of the pivot on frame {W} according to the changes in the CoP during walking. Based on the state shown in Figure 16, at *t* = *n* − 1 (Figure 16a), the CoP of the left leg was used as the pivot (Equation (12)). Subsequently, as the position of the CoP was calculated on frame {8}, the position vector of the CoP on frame {W} was calculated through a homogeneous transformation from kinematic analysis (Equation (13)):

(12) $${}^{8}P_{pivot}^{t}={}^{8}P_{\mathrm{CoP},l}^{t},$$

(13) $${}^{W}P_{pivot}^{t}={}_{B}^{W}T^{t}\cdot {}_{8}^{B}T_{l}^{t}\cdot {}^{8}P_{pivot}^{t}$$

where *P* is a 4 × 1 position vector with its fourth element being 1.

At *t* = *n* (Figure 16b), as the CoP moved because of weight shifting by the wearer during the single stance, the position vector of the pivot on frame {W} was updated considering the same variation as the CoP. The variation in the CoP was calculated by comparing the position vector of the current CoP (${}^{8}P_{\mathrm{CoP},l}^{t}$) and previous CoP (${}^{8}P_{\mathrm{CoP},l}^{t-1}$) on frame {B}. The calculated variation was also transformed to the vector on frame {W} ($\Delta {}^{W}P_{pivot}^{t}$) through homogeneous transformation; it was used for updating the current pivot position vector by adding it to the previous pivot position vector as follows:

(14) $$\Delta {}^{W}P_{pivot}^{t}={}_{B}^{W}T^{t}\cdot ({}^{B}P_{pivot}^{t}-{}^{B}P_{pivot}^{t-1|t}),$$

(15) $${}^{W}P_{pivot}^{t}={}^{W}P_{pivot}^{t-1}+\Delta {}^{W}P_{pivot}^{t},$$

where ${}^{B}P_{pivot}^{t}$ and ${}^{B}P_{pivot}^{t-1|t}$ are ${}_{8}^{B}T_{l}^{t}\cdot {}^{8}P_{\mathrm{CoP},l}^{t}$ and ${}_{8}^{B}T_{l}^{t}\cdot {}^{8}P_{\mathrm{CoP},l}^{t-1}$, respectively, and indicate the position vector of the pivots calculated with respect to frame {B} at time *t*.

At *t* = *n* + 1 (Figure 16c), i.e. double stance, the supporting leg changed from the left leg to the right leg; the CoP of the left foot was used as the position vector of the previous pivot, and that of the right foot was used as the position vector of the current pivot as follows:

(16) $${}^{B}P_{pivot}^{t}={}_{8}^{B}T_{r}^{t}\cdot {}^{8}P_{\mathrm{CoP},r}^{t}\;\mathrm{and}\;{}^{B}P_{pivot}^{t-1|t}={}_{8}^{B}T_{l}^{t}\cdot {}^{8}P_{\mathrm{CoP},l}^{t},$$

The variation in the CoP on frame {W} was calculated using Equation (14), and the position vector of the current pivot was also updated using Equation (15). While the exoskeleton walked on the ground, the position vector of the pivot was continually updated to calculate the exoskeleton motion for frame {W} through this process.

#### 2.2.4. Modeling the Contact Surface as a Plane

The calculated position vectors of the pivot are the contact points on the walking ground. Therefore, we used the pivots as markers representing the geometry of the ground. Only the pivot collected in the FF phase was utilized as a marker for terrain estimation because it was fully in contact with the ground. The markers were separately grouped for each step. The set *S_N,m_* consisting of *m* markers collected for the *N*th step is expressed as follows:

(17) $$S_{N,m}=\{{}^{W}P_{pivot}^{t-m+1},{}^{W}P_{pivot}^{t-m+2},\ldots ,{}^{W}P_{pivot}^{t}\},$$

**Figure 17:**
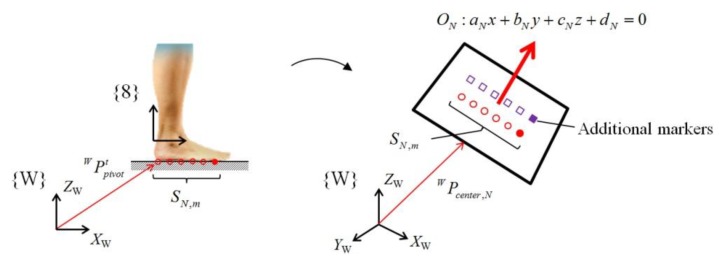
Modelling of the walking ground as a plane using the collected pivot points of each step.

If a sufficient number of markers was accumulated (*m* ≥ 10), the walking ground for the *N*th step was modeled as a spatial plane ($a_{N}x+b_{N}y+c_{N}z+d_{N}=0$) through the least-square method using set *S_N,m_* (Figure 17). However, because the collected points were sufficient to determine a spatial line but insufficient to determine the spatial plane, we added additional virtual markers (purple squares in Figure 17), which were located in the medial direction of the foot and parallel to the calculated CoP, to model the plane. Finally, plane *O_N_*, which was modeled for the *N*th step, is defined as follows:

(18) $$O_{N}=\{u_{N},{}^{W}P_{center,N}^{}\},$$

where $u_{N}$ indicates the normal vector of the modeled plane, and ${}^{W}P_{center,N}^{}$ is the center point of the plane and is the average value of the marker set, *S_N,m_*.

**Figure 18:**
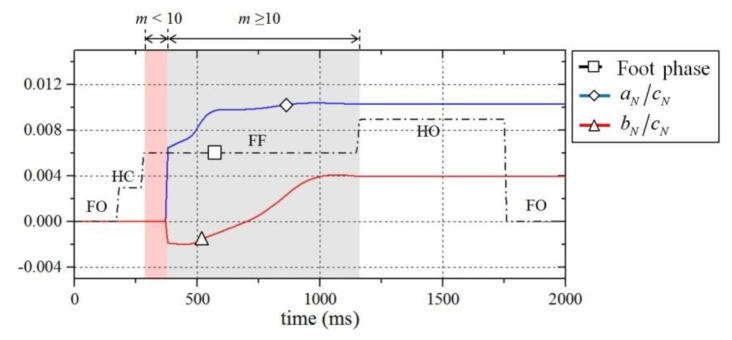
Updating process of the calculated plane coefficients while the exoskeleton is in contact with the ground. The coefficient, *c_N_*, is a constant equal to −1.

Because the markers were continuously collected while the foot of the exoskeleton remained in contact with the ground, the size of the marker set increased during the contact. Therefore, the plane of the ground for each step also changed as the set was updated. Figure 18 shows that the transitions of the plane equation components (*a_N_*, *b_N_*) are continuously updated for one gait cycle during walking. Therefore, the longer the contact with the ground, the closer the modeled plane will be to the actual terrain.

#### 2.2.5. Terrain Feature Estimation

**Figure 19:**
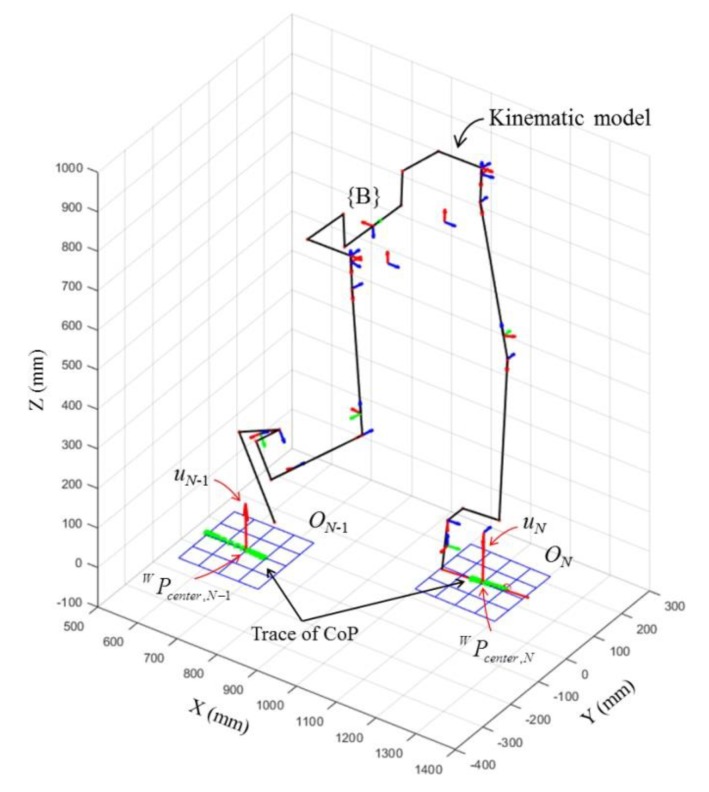
Result of contact surface estimation for both feet during walking.

As the wearer walked on the ground, the contact point sets of both feet were modeled as planes that varied based on the geometry of the ground. Figure 19 shows planes *O_N_* and *O_N_*_− 1_ modeled for steps *N* and *N* − 1, respectively. We calculated the slope and elevation of the walking terrain by comparing the normal vector ($u_{N}$) and center point (${}^{W}P_{center,N}^{}$) of the planes, as follows:

(19) $$\theta_{N}=\mathrm{atan}2([(u_{N}\times Z_{W})\cdot u_{N}],[u_{N}\cdot Z_{W}]),$$

(20) $$h_{N}={}^{W}P_{center,N}^{}(z)-{}^{W}P_{center,N-1}^{}(z),$$

where ${}^{W}P_{center,N}^{}(z)$ indicates component $Z_{W}$ of ${}^{W}P_{center,N}^{}$.

The slope $\theta_{N}$ represents the angle of the normal vector *u_N_* with respect to the *Z*_W_ axis. This value indicates the degree to which the ground is tilted with respect to the direction of gravity. The elevation *h_N_* is the height difference between the floor in the previous step and the floor in the current step. These two features reflect the geometry of the ground; for example, both these features will be low for the level ground case; however, for stairs, the elevation will be high while the slope will be low. In the next section, we show the results of the estimated features of different terrains through walking experiments involving level ground, stairs, and a ramp.

## 3. Experimental Results

**Figure 20:**
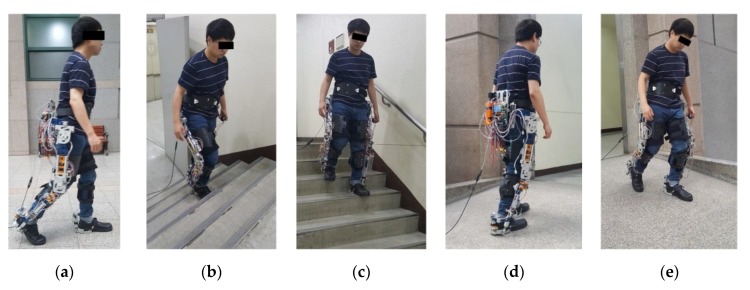
Five types of ambulation tests: ((**a**) level walk (LW), (**b**) stair ascent (SA), (**c**) stair descent (SD), (**d**) ramp ascent (RA), and (**e**) ramp descent (RD)) on three different terrains for performance validation of the proposed method. Ramp ascent and descent were conducted on three ramps with different slopes to validate the performance of the slope estimation.

For evaluating the performance of the proposed method, five types of ambulation tests on different terrains (Figure 20) were performed by involving normal healthy subjects and their demographic characteristics are listed in Table 3. Each subject provided informed consent before participating in the test. The segments and joint axes of the exoskeleton were adjusted to fit to the subject’s body size so that the subject could move comfortably. The experiment began after the subject had sufficient practice to move naturally in the experimental terrain.

**Figure 21:**
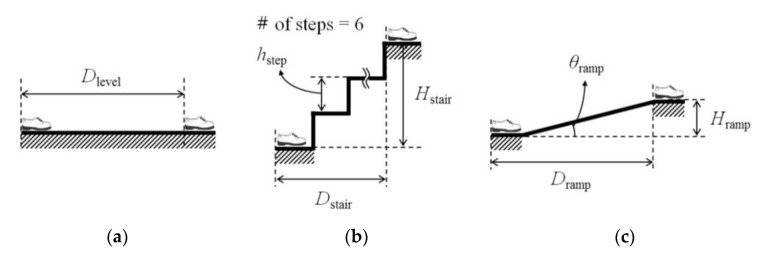
Terrains used for the tests ((**a**) level ground, (**b**)stair, (**c**)ramp). The shoes in the figure indicate the start and end positions of the subject. The height of each step (*h*_step_) of the stairs is 165 mm.

The three types of terrains used in the experiment are shown in Figure 21; the horizontal distance (*D*_case_), vertical distance (*H*_case_), and inclination angle (*θ*_case_) for each terrain are summarized in Table 4. Three ramps with different slopes were used to validate the performance of the slope estimation. It was assumed that the experimental terrains did not change in the lateral direction. The subject began ambulation with the standing posture and then repeated the level walk (LW), stair ascent (SA), stair descent (SD), ramp ascent (RA), and ramp descent (RD) processes on each terrain 10 times. For obtaining consistent experimental results, the start and end positions were marked on the ground as the reference positions for the subject. Sensor data from the exoskeleton were collected at a sampling rate of 100 Hz using a PC. The collected data were analyzed by using MATLAB (MATLAB R2017a, The MathWorks, Inc.).

**Figure 22:**
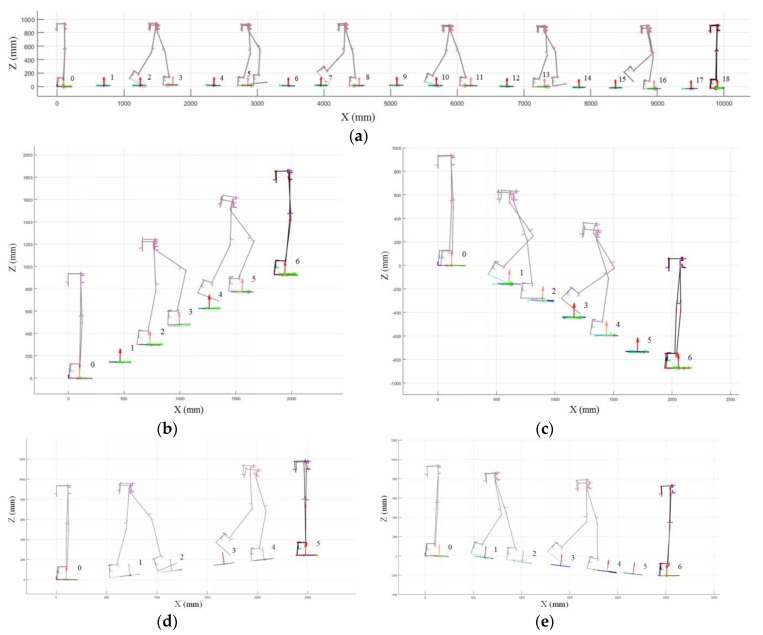
Visualization of position data of the exoskeleton on frame {W} for (**a**) level walk, (**b**) stair ascent, (**c**) stair descent, (**d**) ramp ascent, and (**e**) ramp descent.

**Figure 23:**
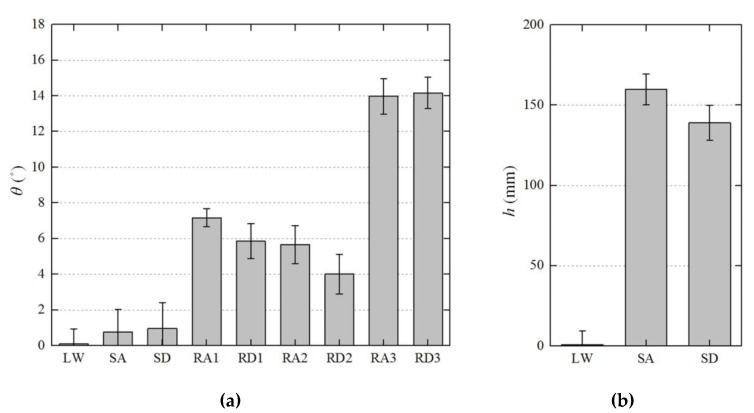
Average (**a**) slope and (**b**) elevation estimation results of all tests for each terrain. The absolute values were used for stairs and ramp descent cases.

Walking data equal for 6510 steps were collected during the experiments for all subjects. Figure 22 shows the kinematic analysis results of the exoskeleton on frame {W} in each terrain and Figure 23 shows the average of the terrain feature estimation results for all subjects. The analysis result in the intermediate process is overlapped in the figure. The estimated planes of each step on the ground on which the exoskeleton walked are depicted with their normal vectors (red arrows). As shown in the results, the method analyzed the exoskeleton movement for each terrain, even though we did not provide any prior knowledge about the experiment terrain. The videos showing the analysis results of the exoskeleton on the different terrains can be found in Supplementary Materials Video S1.

Table 5 summarizes the estimation errors in the total displacement of the subject and the slope and elevation, which were calculated using the proposed method for the experimental terrain. The total accuracy (S_total_) calculated using the entire data was inserted in the last row for each test. The position error per step was calculated by dividing the error between the total calculated displacement of the subject and the actual distance of the experimental section (Table 4) by the number of steps performed for each section. The total root mean square (RMS) error of the horizontal movement displacement in all experiments (*D_RMSE_*) was approximately 13 mm per step, which is approximately 2% of the step length of a normal person, approximately 660 mm [47]. Although the total RMS error (*H_RMSE_*) of the vertical movement displacement was within 10 mm per step except for the SD test, the SD test showed a large error of approximately 20 mm per step. This is because, in the SD motion, the front of the flexible foot touches the ground first, instead of the rigid heel cuff at each step. Therefore, if an accurate human foot model is used to calculate the position of the accurate contact point, the error can be minimized. The lateral direction error (*Y_RMSE_*) reflects the degree of the drift effect caused by the AHRS. The error was resulted lower than 10 mm/step for the total walking distance in the experiments on the level, the ramps 2 and 3, which is a reasonable result; however, the error with a range of 10–20 mm per step was observed in the experiments on the stairs and the ramp 1.

Among the estimation results for each terrain, the RMS error ($\theta_{RMSE}$) of the slope was less than 2° for all tests. The estimated RMS error of the elevation ($h_{RMSE}$) was calculated for the LW, SA, and SD tests only, as the stride of the subject was not limited in the RA and RD tests. The estimation error of the elevation in the LW test was found to be within approximately 10 mm; and, the errors in the SA and SD tests were approximately 7 % and 17% of 165 mm which is the height of the experimental staircase, respectively. For the SD test, there was a larger error than the other experiments because the front part of the foot that is allowed to bent touched the ground first.

On average, the errors of the proposed method for the entire terrain in each direction were determined as 10.29, 9.45, and 9.22 mm per step, respectively; the terrain feature estimation result showed that the RMS errors of the slope and elevation were approximately 1.2° and 10% of the height of the stairs, respectively. Consequently, we could obtain best results in the level ground experiment, but the errors in the stair and ramp experiments need to be minimized in future. In the next section, we discuss directions to reduce these errors.

**Figure 24:**
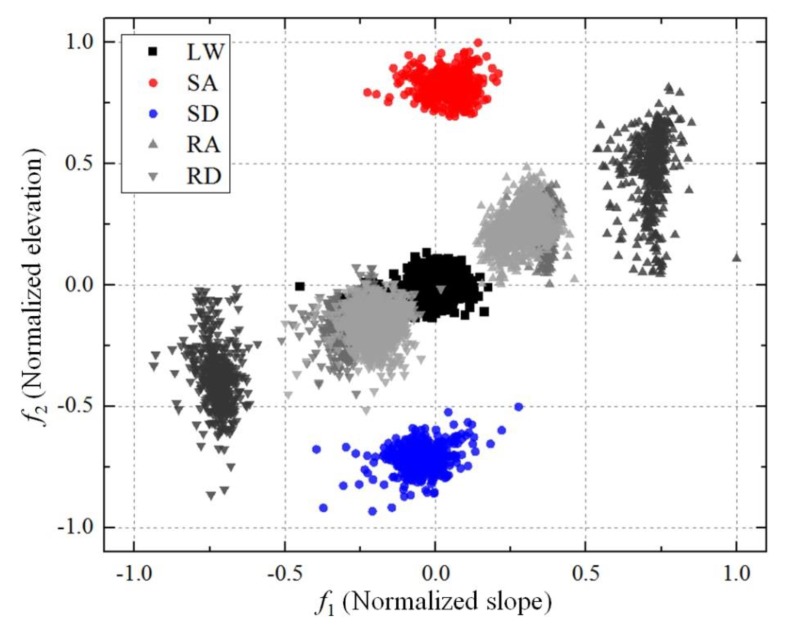
Normalized terrain slope and elevation results in feature space. The maximum values of $\theta_{N}$ and $h_{N}$ were used for normalization. The samples of RA and RD for different slopes are colored differently (light gray: ramp 1, medium gray: ramp 2, dark gray: ramp 3).

The terrain features of each step—slope and elevation—, which were estimated using the proposed method, can also be used for classifying the locomotion mode. We normalized the slope and elevation results of 6510 steps in total by dividing them by their maximum values; we then used the normalized values as features to classify the walking terrain. Figure 24 shows the distribution of the samples obtained using the selected features in the feature space. In the figure, SA and SD test samples are clearly distinguished from other samples, but the samples of RA and RD test on low slopes are partially mixed with the samples of LW test. Table 6 presents the classification performance using a support vector machine (SVM) for the total dataset of steps, and Table 7 shows the classification performance on each subject. Classification using the estimated features showed recognition accuracy higher than 95% for all experimental motions and for all subjects. From these results, we confirmed that the terrain features estimated using the proposed method can be used for classifying the walking movements.

## 4. Discussion

In this paper, we proposed a method to estimate the terrain features, namely the slope and elevation, of the walking ground by finding the contact surface based on the kinematic analysis of the lower limb exoskeleton. The proposed method was evaluated via experiments involving three types of terrains: level walk for 10 m, stair ascent and descent, and ramp ascent and descent with three different slopes. In the experimental results, except for SD walking, the total displacement calculation error for all directions was approximately 10 mm per step, which corresponds to 1.5% of 660 mm, which is the step length of a normal person; the slope and elevation estimation errors of the ground were approximately 0.8 to 1.8° and 8 to 28 mm, respectively. These error values also show how roughly the motion of the exoskeleton is being calculated. In addition, the classification results obtained using estimated features for the five walking movements showed recognition accuracy higher than 95%; this indicates that the terrain features estimated using the proposed method can be used as features for classification.

The main contribution of this study is that the proposed method can estimate information about unknown terrains using the sensors embedded in the exoskeleton only, without direct measurements of the surrounding terrain. This advantage allows the system to determine the surrounding terrain and control it appropriately in unpredicted and unknown environments, rather than being confined to specific environments. In addition, by identifying the specific slope and elevation of the walking terrain, it can be used to support the wearer’s walking motion safely, by performing stability evaluation or trajectory generation for the next step. Additionally, as this study estimated the terrain features by using only the sensors embedded in the general exoskeleton, it is expected that this method can be applied to other exoskeleton systems without difficulty.

The updating process of the modeled plane is an important advantage of our method. The excellent and comprehensive work demonstrated by Huo et al. [48] used terrain features for the gait mode detection of a lower limb exoskeleton. In their reported results, they estimated terrain features with the following errors of mean = 6 mm, std = 34 mm in LW; mean = 1 mm, std = 18 mm in SA and SD for the elevation; mean = 0.37°, std = 2° in LW, SA and SD for the slope. In our study, the estimated terrain features with the following errors of: mean = 0.93 mm, std = 8.55 mm for LW; mean = 5.16 mm, std = 9.73 mm for SA; mean = 25.9 mm, std = 10.9 mm for SD for the elevation; and mean = 0.03°, std = 1.15° for LW, SA and SD for the slope. Although, the mean value of their stairs height estimation result is better than the result of ours, our method shows lower standard deviations in all cases. Therefore, the updating process that continuously improves the surface vectors while the foot is in contact with the ground is expected to further refine the estimation performance.

However, the proposed method still has challenges to overcome. First, as terrain information is estimated after the wearer steps on the ground, the terrain for the next step cannot be estimated before contact. However, the prediction of the next terrain can be complemented using a combination of the nature of the walking cycle [20], which is usually repeated for the next few steps after the first step, and a user intention estimation algorithm. The drift error from the AHRS also needs to be reduced. In the experiments, we limited the test time to minimize the effect of the drift error. Generally, the drift error can be corrected by a magnetometer; however, it cannot be used for robotic applications because of the magnetic distortions caused by electrical actuators and the indoor environment, such as steel window frames or handrails on stairs. Therefore, in future studies, a more elaborate filter, such as the ZUPT algorithm [49] and the model-based extended Kalman filter [35,50], or an additional sensor should be considered to predict the gyroscope bias and correct drift errors. Finally, the exact position of the contact point between the exoskeleton and ground should be calculated. In particular, this issue was apparently identified during the SD test in our study. We configured the front part of the foot to be bent for the smooth walking motion of the wearer, but the largest error was found in the SD test owing to the absence of the model for this part. This position error can be reduced by utilizing the roll-over model of the human foot for calculating the position of the contact point.

In this study, because the exoskeleton was used without any actuation modules, the subjects had to move their limbs by themselves. However, the weight of the exoskeleton did not significantly affect the normal subjects because only 1 female subject who had asked for a rest for 15 min in the test which was performed in an average of more than three hours. But, controlling the joints of the exoskeleton using the actuation modules should be developed to assist safely the motion of the people with muscular weakness in the future study.

**Figure 25:**
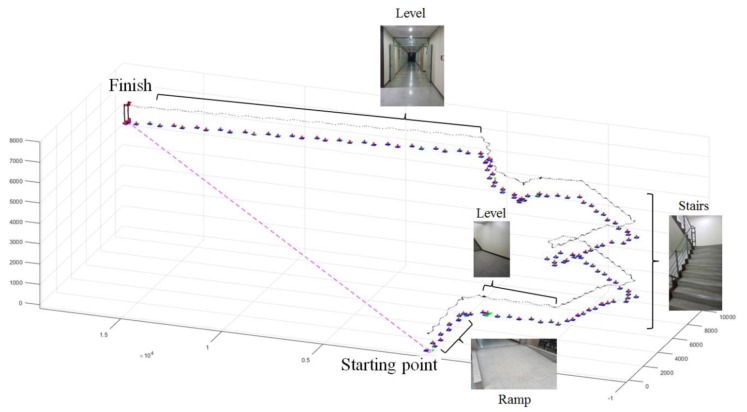
Test result for the motion of the exoskeleton in a building environment. (The video showing the analysis results of this test can be found in Supplementary Materials Video S2).

Additionally, utilizing the exoskeleton system, which consists of the modular actuators used in this study, in various modes according to the purpose of the user in various applications is a future challenge. As part of this work, we confirmed the possibility of using the developed exoskeleton as a system for motion capture of the wearer. Figure 25 shows the estimation results for the wearer’s position obtained when the wearer wore the exoskeleton inside a building. Through this result, we verified that our exoskeleton could be used to estimate the wearer’s position in a hospital or outdoor environment by overcoming the drawbacks of the vision-based motion capture system, which can only be used in a limited space, and global positioning systems, which cannot estimate positions in a building. The other objective for future work is that will focus on applying our method from this study to modify the gait trajectory on various terrains in real-time.

In conclusion, we developed a method to estimate the slope and elevation of walking terrains based on the kinematic analysis of the lower limb exoskeleton by finding the contact surface with the terrain. We verified the position and terrain feature estimation accuracies of the proposed method through experiments on different terrains involving level walking for 10 m, stair ascent and descent, and ramp ascent and descent. The proposed method approximately analyzed the movement of the exoskeleton on various terrains even though no prior information on the walking terrain was provided. The method is expected to enable exoskeleton systems to actively assist walking in various environments including unrestricted daily living environments.

## Figures and Tables

**Table 1:** Joint ranges of motion of a human and the exoskeleton.

Joint Motion	ROM (°)	In Walking (°)	Exoskeleton (°)
Hip flexion/extension	120/30	36/6	110/25
Hip adduction/abduction	35/40	7/6	20/30
Hip medial/lateral rotation	30/60	10/13	20/20
Knee extension/flexion	10/140	0/64	10/110
Ankle dorsi/plantar flexion	20/50	11/19	20/20
Ankle inversion/eversion	35/20	5/7	10/10

**Table 2:** D-H parameters for the kinematic analysis. The variables are depicted in Figure 2, Figure 3 and Figure 4.

Frame	Left Leg				Right Leg			
*i*	*α_i_*_− 1_ (rad)	*a_i_*_− 1_ (mm)	*d_i_* (mm)	*θ_i_* (rad)	*α_i_*_− 1_ (rad)	*a_i_*_− 1_ (mm)	*d_i_* (mm)	*θ_i_* (rad)
1	0	*L*_hip,1_	*L*_hip,6_	*θ*_1,*l*_ + (π/2)	0	−*L*_hip,1_	*L*_hip,6_	*θ*_1,*r*_ + (π/2)
2	π/2	*L*_hip,3_	*L*_hip,2_	π/2	π/2	*L*_hip,3_	−*L*_hip,2_	π/2
3	π/2	0	−*L*_hip,4_	*θ*_3,*l*_ + (π/2)	π/2	0	−*L*_hip,4_	*θ*_3,*r*_ + (π/2)
4	π/2	0	0	−π/2	π/2	0	0	−π/2
5	−π/2	*L*_hip,5_	0	*θ*_5,*l*_	−π/2	*L*_hip,5_	0	*θ*_5,*r*_
6	0	*L*_thigh_	0	*θ*_6,*l*_	0	*L*_thigh_	0	*θ*_6,*r*_
7	0	*L*_shank_	−*L*_ankle,2_	*θ*_7,*l*_	0	*L*_shank_	*L*_ankle,2_	*θ*_7,*r*_
8	π/2	0	−*L*_ankle,1_	*θ*_8,*l*_	π/2	0	−*L*_ankle,1_	*θ*_8,*r*_

**Table 3:** Demographic characteristics of the subjects

Subject	Gender	Age (y)	Height (m)	Weight (kg)	BMI (kg/m^2^)	Shoe Size (mm)
S1	Male	33	1.70	68	23.5	260
S2	Male	35	1.68	74	26.2	260
S3	Male	25	1.76	67	21.6	265
S4	Male	29	1.80	74	22.8	270
S5	Male	39	1.76	88	28.4	275
S6	Female	36	1.61	60	23.1	240
S7	Female	43	1.65	70	25.7	250

**Table 4:** Dimensions of the terrains for evaluating the performance of the developed method.

Case	*D*_case_ (mm)	*H*_case_ (mm)	*θ*_case_ (°)
Level	10,000	0	0
Stair	1900	990	0
Ramp 1	12,770	1020	5
Ramp 2	2460	230	6.37
Ramp 3	3600	620	14

**Table 5:** Position error per step and terrain feature estimation error for each test.

		Position Error Per Step			Terrain Estimation Error	
Case	Subject	*D*_RMSE_ (mm)	*H*_RMSE_ (mm)	*Y*_RMSE_ (mm)	*θ*_RMSE_ (°)	*h*_RMSE_ (mm)
LW	S1	6.15	4.18	5.93	1.30	8.90
	S2	4.83	4.02	5.35	0.71	6.36
	S3	2.45	2.78	3.41	0.66	11.43
	S4	2.11	6.67	6.77	1.10	8.01
	S5	1.94	1.93	4.51	0.84	8.33
	S6	10.89	5.57	3.82	0.76	8.81
	S7	4.00	6.14	5.46	0.69	7.38
	**S_total_**	**5.38**	**4.70**	**5.11**	**0.86**	**8.60**
SA	S1	4.94	11.23	12.22	1.20	13.06
	S2	8.48	1.90	12.75	1.75	9.62
	S3	6.04	8.98	18.24	1.23	12.38
	S4	17.70	8.10	16.83	1.51	10.65
	S5	7.98	8.81	18.59	1.79	12.54
	S6	19.04	4.77	11.45	1.52	7.93
	S7	10.89	3.44	10.41	1.32	10.08
	**S_total_**	**12.05**	**7.42**	**14.63**	**1.48**	**11.00**
SD	S1	7.46	24.12	17.92	1.66	26.28
	S2	5.18	14.02	15.75	1.95	21.72
	S3	8.79	20.59	10.62	1.41	27.09
	S4	12.53	26.02	9.26	1.32	33.04
	S5	4.62	19.90	8.70	2.64	26.51
	S6	7.50	24.01	13.36	1.59	32.49
	S7	12.14	19.48	15.98	1.26	27.57
	**S_total_**	**8.89**	**21.55**	**13.51**	**1.74**	**28.12**
RA1	S1	11.78	8.81	14.80	1.09	-
	S2	15.84	13.89	7.82	0.87	-
	S3	16.13	6.06	11.25	0.83	-
	S4	12.47	2.73	9.19	1.03	-
	S5	8.40	9.78	6.66	1.04	-
	S6	11.01	14.26	9.82	0.91	-
	S7	4.39	2.88	9.33	0.91	-
	**S_total_**	**11.96**	**9.49**	**9.94**	**0.94**	-
RD1	S1	7.97	9.04	21.74	1.13	-
	S2	13.98	7.73	9.15	1.14	-
	S3	10.25	2.35	8.69	0.63	-
	S4	9.59	2.74	10.45	1.30	-
	S5	9.40	3.55	5.91	1.13	-
	S6	11.47	5.40	8.23	1.06	-
	S7	8.66	5.02	7.91	1.31	-
	**S_total_**	**10.37**	**5.50**	**10.99**	**1.11**	-
RA2	S1	4.47	14.84	5.21	1.05	-
	S2	2.66	10.46	5.18	1.33	-
	S3	2.45	5.90	3.07	1.15	-
	S4	13.81	6.87	4.62	1.55	-
	S5	13.06	3.84	6.40	1.32	-
	S6	10.21	17.86	4.35	1.14	-
	S7	11.78	3.31	7.83	1.19	-
	**S_total_**	**9.73**	**10.49**	**5.41**	**1.25**	-
RD2	S1	5.44	15.05	3.82	1.60	-
	S2	7.28	10.71	2.99	1.54	-
	S3	11.52	6.14	4.38	1.40	-
	S4	11.09	1.84	9.16	1.60	-
	S5	14.14	5.70	6.31	1.49	-
	S6	7.98	10.36	8.74	1.37	-
	S7	14.61	4.47	3.80	1.42	-
	**S_total_**	**11.03**	**8.37**	**6.19**	**1.50**	-
RA3	S1	8.29	6.57	7.48	1.70	-
	S2	17.91	12.46	9.76	0.85	-
	S3	14.18	11.28	6.13	1.23	-
	S4	15.32	4.33	14.95	0.84	-
	S5	14.35	5.85	6.25	0.99	-
	S6	6.53	13.76	5.94	0.62	-
	S7	8.38	9.97	8.37	0.62	-
	**S_total_**	**12.74**	**9.77**	**8.99**	**0.99**	-
RD3	S1	10.38	4.94	8.37	0.78	-
	S2	8.86	3.82	7.01	1.39	-
	S3	9.07	5.06	6.02	0.93	-
	S4	9.93	6.81	11.92	0.59	-
	S5	11.42	5.53	5.73	0.60	-
	S6	8.44	7.40	6.89	0.95	-
	S7	13.59	14.25	9.46	0.75	-
	**S_total_**	**10.43**	**7.77**	**8.17**	**0.89**	-

The total accuracy (S_total_) calculated using entire subject data for each case is represented in bold type.

**Table 6:** Confusion matrix and classification performance of the results using SVM.

		Predicted					Performance		
		LW	SA	SD	RA	RD	Precision	Recall	F1-score
Actual	LW	1770	0	0	1	24	0.990	0.986	0.988
	SA	0	426	0	0	0	1.000	1.000	1.000
	SD	0	0	419	0	1	1.000	0.998	0.999
	RA	1	0	0	1879	0	0.999	0.999	0.999
	RD	17	0	0	0	1972	0.987	0.991	0.989

**Table 7:** Classification performance (F1-score) for each subject.

Case	S1	S2	S3	S4	S5	S6	S7
LW	0.953	0.984	0.985	0.995	0.996	1.000	0.998
SA	1.000	0.981	1.000	1.000	1.000	1.000	1.000
SD	1.000	1.000	1.000	1.000	1.000	1.000	1.000
RA	1.000	0.995	1.000	0.998	1.000	1.000	1.000
RD	0.962	0.983	0.985	0.998	0.997	1.000	0.998

## Supplementary Materials

The following are available online at https://www.mdpi.com/1424-8220/19/20/4418/s1, Video S1: Kinematic analysis results of the exoskeleton for level walk, stair ascent, stair descent, ramp ascent, and ramp descent, Video S2: Kinematic analysis result for the motion of the exoskeleton in a building environment.

## References

[1] Pons J.L. (2008). Wearable Robots: Biomechatronic Exoskeletons.

[2] Young A.J., Ferris D.P. (2017). State-of-the-Art and Future Directions for Robotic Lower Limb Robotic Exoskeletons. IEEE Trans. Neural Syst. Rehabil. Eng..

[3] Aliman N., Ramli R., Haris S.M. (2017). Design and Development of Lower Limb Exoskeletons: A Survey. Rob. Auton. Syst..

[4] De Looze M.P., Bosch T., Krause F., Stadler K.S., O’Sullivan L.W. (2016). Exoskeletons for Industrial Application and Their Potential Effects on Physical Work Load. Ergonomics.

[5] Herr H. (2009). Exoskeletons and Orthoses: Classification, Design Challenges and Future Directions. J. Neuroeng. Rehabil..

[6] Gorgey A.S. (2018). Robotic Exoskeletons: The Current Pros and Cons. World J. Orthop..

[7] Meng W., Liu Q., Zhou Z., Ai Q., Sheng B., Xie S.S. (2015). Recent Development of Mechanisms and Control Strategies for Robot-Assisted Lower Limb Rehabilitation. Mechatronics.

[8] Tucker M.R., Olivier J., Pagel A., Bleuler H., Bouri M., Lambercy O., Millán J Del R., Riener R., Vallery H., Gassert R. (2015). Control Strategies for Active Lower Extremity Prosthetics and Orthotics: A Review. J. Neuroeng. Rehabil..

[9] Du L., Zhang F., Liu M., Huang H. (2012). Toward Design of an Environment-Aware Adaptive Locomotion-Mode-Recognition System. IEEE Trans. Biomed. Eng..

[10] CYBATHLON, Races and Disciplines, Powered Exoskeleton Race. https://cybathlon.ethz.ch/races-and-disciplines/powered-exoskeleton-race.html.

[11] Liu M., Wang D., Huang H.H. (2016). Development of an Environment-Aware Locomotion Mode Recognition System for Powered Lower Limb Prostheses. IEEE Trans. Neural Syst. Rehabil. Eng..

[12] Laschowski B., McNally W., Wong A., McPhee J. Preliminary Design of an Environment Recognition System for Controlling Robotic Lower-Limb Prostheses and Exoskeletons. Proceedings of the 2019 IEEE International Conference on Rehabilitation Robotics.

[13] Xu F., Lin X., Cheng H., Huang R., Chen Q. Adaptive Stair-Ascending and Stair-Descending Strategies for Powered Lower Limb Exoskeleton. Proceedings of the 2017 IEEE International Conference on Mechatronics and Automation.

[14] Scandaroli G.G., Borges G.A., Ishihara J.Y., Terra M.H., da Rocha A.F., de Oliveira Nascimento F.A. Estimation of Foot Orientation with Respect to Ground for an above Knee Robotic Prosthesis. Proceedings of the 2009 IEEE/RSJ International Conference on Intelligent Robots and Systems.

[15] Li Q., Young M., Naing V., Donelan J.M. Walking Speed and Slope Estimation Using Shank-Mounted Inertial Measurement Units. Proceedings of the IEEE 11th International Conference on Rehabilitation Robotics.

[16] Lawson B.E., Varol H.A., Goldfarb M. (2011). Standing Stability Enhancement with an Intelligent Powered Transfemoral Prosthesis. IEEE Trans. Biomed. Eng..

[17] Zhu A., Li Y., Wu Y., Wu M., Zhang X. Locomotion Mode Recognition Based on Foot Posture and Ground Reaction Force. Proceedings of the 15th International Conference on Ubiquitous Robots.

[18] Kyeong S., Shin W., Yang M., Heo U., Feng J., Kim J. (2019). Recognition of Walking Environments and Gait Period by Surface Electromyography. Front. Inf. Technol. Electron. Eng..

[19] Au S., Berniker M., Herr H. (2008). Powered Ankle-Foot Prosthesis to Assist Level-Ground and Stair-Descent Gaits. Neural Netw..

[20] Ronsse R., Lenzi T., Vitiello N., Koopman B., van Asseldonk E., De Rossi S.M.M., van den Kieboom J., van der Kooij H., Carrozza M.C., Ijspeert A.J. (2011). Oscillator-Based Assistance of Cyclical Movements: Model-Based and Model-Free Approaches. Med. Biol. Eng. Comput..

[21] Schiele A., van der Helm F.C.T. (2006). Kinematic Design to Improve Ergonomics in Human Machine Interaction. IEEE Trans. Neural Syst. Rehabil. Eng..

[22] Wu G., Siegler S., Allard P., Kirtley C., Leardini A., Rosenbaum D., Whittle M., D’Lima D.D., Cristofolini L., Witte H. (2002). ISB Recommendation on Definitions of Joint Coordinate System of Various Joints for the Reporting of Human Joint Motion—Part I: Ankle, Hip, and Spine. J. Biomech..

[23] Bartenbach V., Wyss D., Seuret D., Riener R. A Lower Limb Exoskeleton Research Platform to Investigate Human-Robot Interaction. Proceedings of the 2015 IEEE International Conference on Rehabilitation Robotics.

[24] Beil J., Marquardt C., Asfour T. Self-Aligning Exoskeleton Hip Joint: Kinematic Design with Five Revolute, Three Prismatic and One Ball Joint. Proceedings of the 2017 International Conference on Rehabilitation Robotics.

[25] Rosen J., Perry J.C., Manning N., Burns S., Hannaford B. The Human Arm Kinematics and Dynamics during Daily Activities-Toward a 7 DOF Upper Limb Powered Exoskeleton. Proceedings of the 12th International Conference on Advanced Robotics.

[26] Yang W., Yang C.-J., Wei Q.X. Design of an Anthropomorphic Lower Extremity Exoskeleton with Compatible Joints. Proceedings of the 2014 IEEE International Conference on Robotics and Biomimetics.

[27] Hill P.F., Vedi V., Williams A., Iwaki H., Pinskerova V., Freeman M.A. (2000). Tibiofemoral Movement 2: The Loaded and Unloaded Living Knee Studied by MRI. J. Bone Jt. Surg. Br..

[28] Komistek R.D., Dennis D.A., Mahfouz M. (2003). In Vivo Fluoroscopic Analysis of the Normal Human Knee. Clin. Orthop. Relat. Res..

[29] Blankevoort L., Huiskes R., de Lange A. (1988). The Envelope of Passive Knee Joint Motion. J. Biomech..

[30] Brockett C.L., Chapman G.J. (2016). Biomechanics of the Ankle. Orthop. Trauma.

[31] Ren L., Jones R.K., Howard D. (2007). Predictive Modelling of Human Walking over a Complete Gait Cycle. J. Biomech..

[32] Srinivasan S., Raptis I.A., Westervelt E.R. (2008). Low-Dimensional Sagittal Plane Model of Normal Human Walking. J. Biomech. Eng..

[33] The 7th Survey of the Korean Body Size. https://sizekorea.kr/page/report/1.

[34] Cheng P., Oelmann B. (2010). Joint-Angle Measurement Using Accelerometers and Gyroscopes-A Survey. IEEE Trans. Instrum. Meas..

[35] Cantelli L., Muscato G., Nunnari M., Spina D. (2015). A Joint-Angle Estimation Method for Industrial Manipulators Using Inertial Sensors. IEEE/ASME Trans. Mechatron..

[36] Brennan A., Zhang J., Deluzio K., Li Q. (2011). Quantification of Inertial Sensor-Based 3D Joint Angle Measurement Accuracy Using an Instrumented Gimbal. Gait Posture.

[37] Wang Y., Chen W., Tomizuka M. (2013). Extended Kalman Filtering for Robot Joint Angle Estimation Using MEMS Inertial Sensors. IFAC Proc. Vol..

[38] Kim J. H., Shim M., Ahn D. H., Son B. J., Kim S. Y., Kim D. Y., Baek Y. S., Cho B. K. (2015). Design of a Knee Exoskeleton Using Foot Pressure and Knee Torque Sensors. Int. J. Adv. Robot. Syst..

[39] Pataky T.C., Mu T., Bosch K., Rosenbaum D., Goulermas J.Y. (2011). Gait Recognition: Highly Unique Dynamic Plantar Pressure Patterns among 104 Individuals. J. R. Soc. Interface.

[40] Hessert M.J., Vyas M., Leach J., Hu K., Lipsitz L.A., Novak V. (2005). Foot Pressure Distribution during Walking in Young and Old Adults. BMC Geriatr..

[41] Bartenbach V., Gort M., Riener R. Concept and Design of a Modular Lower Limb Exoskeleton. Proceedings of the 6th IEEE RAS/EMBS International Conference on Biomedical Robotics and Biomechatronics.

[42] dos Santos W.M., Nogueira S.L., de Oliveira G.C., Peña G.G., Siqueira A.A.G. Design and Evaluation of a Modular Lower Limb Exoskeleton for Rehabilitation. Proceedings of the 2017 International Conference on Rehabilitation Robotics.

[43] Grosu V., Rodriguez-Guerrero C., Grosu S., Vanderborght B., Lefeber D. (2017). Design of Smart Modular Variable Stiffness Actuators for Robotic-Assistive Devices. IEEE/ASME Trans. Mechatron..

[44] Cho S. H., Park J. M., Kwon O. Y. (2004). Gender Differences in Three Dimensional Gait Analysis Data from 98 Healthy Korean Adults. Clin. Biomech..

[45] Giovacchini F., Vannetti F., Fantozzi M., Cempini M., Cortese M., Parri A., Yan T., Lefeber D., Vitiello N. (2015). A Light-Weight Active Orthosis for Hip Movement Assistance. Rob. Auton. Syst..

[46] Craig J.J. (2005). Introduction to Robotics, Mechanics and Control.

[47] Bovi G., Rabuffetti M., Mazzoleni P., Ferrarin M. (2011). A Multiple-Task Gait Analysis Approach: Kinematic, Kinetic and EMG Reference Data for Healthy Young and Adult Subjects. Gait Posture.

[48] Huo W., Mohammed S., Amirat Y., Kong K. (2018). Fast Gait Mode Detection and Assistive Torque Control of an Exoskeletal Robotic Orthosis for Walking Assistance. IEEE Trans. Robot..

[49] Wu X., Wang Y., Pottie G. A Non-ZUPT Gait Reconstruction Method for Ankle Sensors. Proceedings of the 2014 36th Annual International Conference of the IEEE Engineering in Medicine and Biology Society.

[50] Lee M.S., Ju H., Song J.W., Park C.G. (2015). Kinematic Model-Based Pedestrian Dead Reckoning for Heading Correction and Lower Body Motion Tracking. Sensors.

